# Lessons Learned Comparing Immune System Alterations of Bacterial Sepsis and SARS-CoV-2 Sepsis

**DOI:** 10.3389/fimmu.2020.598404

**Published:** 2020-11-30

**Authors:** Xijie Dong, Chuntao Wang, Xinghua Liu, Wei Gao, Xiangjun Bai, Zhanfei Li

**Affiliations:** Trauma Center, Department of Emergency and Traumatic Surgery, Tongji Hospital of Tongji Medical College, Huazhong University of Science and Technology, Wuhan, China

**Keywords:** COVID-19, sepsis, lymphocyte subsets, cytokine storm, immunoglobulin

## Abstract

**Background:**

Bacterial sepsis has been used as a prototype to understand the pathogenesis of severe coronavirus disease 2019 (COVID-19). In addition, some management programs for critically ill COVID-19 patients are also based on experience with bacterial sepsis. However, some differences may exist between these two types of sepsis.

**Methods:**

This retrospective study investigated whether there are differences in the immune system status of these two types of sepsis. A total of 64 bacterial sepsis patients and 43 patients with severe acute respiratory syndrome coronavirus 2 (SARS-CoV-2) sepsis were included in this study. Demographic data were obtained from medical records. Laboratory results within 24 h after the diagnosis of sepsis were provided by the clinical laboratory.

**Results:**

The results of blood routine (neutrophil, lymphocyte, and monocyte counts), infection biomarkers (C-reactive protein, ferritin, and procalcitonin levels), lymphocyte subset counts (total T lymphocyte, CD4+ T cell, CD8+ T cell, B cell, and NK cell counts), and lymphocyte subset functions (the proportions of PMA/ionomycin-stimulated IFN-γ positive cells in CD4+, CD8+ T cells, and NK cells) were similar in bacterial sepsis patients and SARS-CoV-2 sepsis patients. Cytokine storm was milder, and immunoglobulin and complement protein levels were higher in SARS-CoV-2 sepsis patients.

**Conclusions:**

There are both similarities and differences in the immune system status of bacterial sepsis and SARS-CoV-2 sepsis. Our findings do not support blocking the cytokine storm or supplementing immunoglobulins in SARS-CoV-2 sepsis, at least in the early stages of the disease. Treatments for overactivation of the complement system and lymphocyte depletion may be worth exploring further.

## Introduction

Coronavirus disease 2019 (COVID-19) has become a global pandemic. As of the submission of this study, about 38 million infections have been confirmed and approximately 1 million deaths have been reported. These shocking numbers are still growing at an alarming rate. Since the outbreak of the epidemic, huge efforts have been made to deal with the challenges of the virus. Among these, clinicians have focused their attention on how to improve the prognosis of critically ill COVID-19 patients.

In clinical practice, it has been noticed that many critically ill COVID-19 patients developed severe organ dysfunction due to dysregulated response to infection and met the diagnostic criteria for sepsis (1–5). In such cases, most clinicians use bacterial sepsis as a prototype to understand the pathogenesis of severe COVID-19, and some management protocols for critically ill COVID-19 patients are also based on experience with bacterial sepsis (3, 6). However, unlike the sepsis that we often refer to mostly caused by bacterial infection, COVID-19 sepsis is a viral sepsis caused by severe acute respiratory syndrome coronavirus 2 (SARS-CoV-2) infection (7). Some differences may exist between these two types of sepsis. For instance, it has been reported that quick Sequential Organ Failure Assessment (qSOFA) was not appropriate to identify COVID-19 patients who will experience poor outcomes typical of sepsis. mHLA-DR expression levels have also been reported to be higher and plasma IL-6 levels were lower in SARS-CoV-2 sepsis patients (8–11). These differences allow us to question whether the changes in the immune system induced by SARS-CoV-2 sepsis differ from those caused by bacterial sepsis and whether it is appropriate to translate treatment recommendations from bacterial sepsis.

The optimal management of sepsis patients requires a clear understanding of alterations in immune system. In this study, we compared changes observed in the immune system of bacterial sepsis patients and SARS-CoV-2 sepsis patients. We also provide recommendations concerning appropriate immunotherapy for SARS-CoV-2 sepsis based on these comparisons.

## Methods

### Study Design and Patients

This retrospective study was performed at a university-affiliated hospital designated by the government as a center for the treatment of severe COVID-19 patients in Wuhan. Approval was obtained from the Medical Ethics Committee of the hospital with a waiver of informed consent.

Sepsis was defined as life-threatening organ dysfunction caused by a dysregulated host response to infection (2). Bacterial sepsis patients with pulmonary infection were defined as the bacterial sepsis group, and viral sepsis patients with SARS-CoV-2 infection were defined as the SARS-CoV-2 sepsis group in our study. The exclusion criteria were patients less than 18 years old; with autoimmune diseases; severe systemic inflammation caused by other diseases; or chronic diseases requiring immunomodulation therapy. Patients with incomplete data were withdrawn from the study. Clinical characteristics that likely modify immune function were almost exactly matched in the two groups (age, sex, and chronic medical illness).

### Data Collection

Demographic data, medical history, the SOFA score, and the Acute Physiology and Chronic Health Evaluation (APACHE) II score were obtained from the clinical information systems. The results of blood routine, infection biomarkers, cytokines, immunoglobulins, complement proteins, as well as lymphocyte subset counts and functions, were detected and reported by the clinical laboratory. Lymphocyte subset functions were evaluated by the proportions of PMA/ionomycin-stimulated IFN-γ positive cells in CD4+, CD8+ T cells, and NK cells. Flow cytometry analysis was performed as previously described (12). SOFA score, APACHE II score, and all laboratory results represent the data obtained within 24 h of the diagnosis of sepsis.

### Statistical Analysis

Continuous and categorical variables are presented as median (interquartile range) and number (percentage) in this study. Comparisons of two independent samples were performed using the Wilcoxon rank-sum test (for non-normally distributed data), chi-square tests (for unordered categorical data) or Fisher exact probability test (for unordered categorical data). Statistical analyses and graphics were developed using SPSS v26.0 (IBM, Armonk, NY) and GraphPad Prism v8.3.0 (GraphPad Software, San Diego, CA). A two-sided *p*-value of less than 0.05 was considered statistically significant.

## Results

### Cohort Characteristics

**Figure 1:**
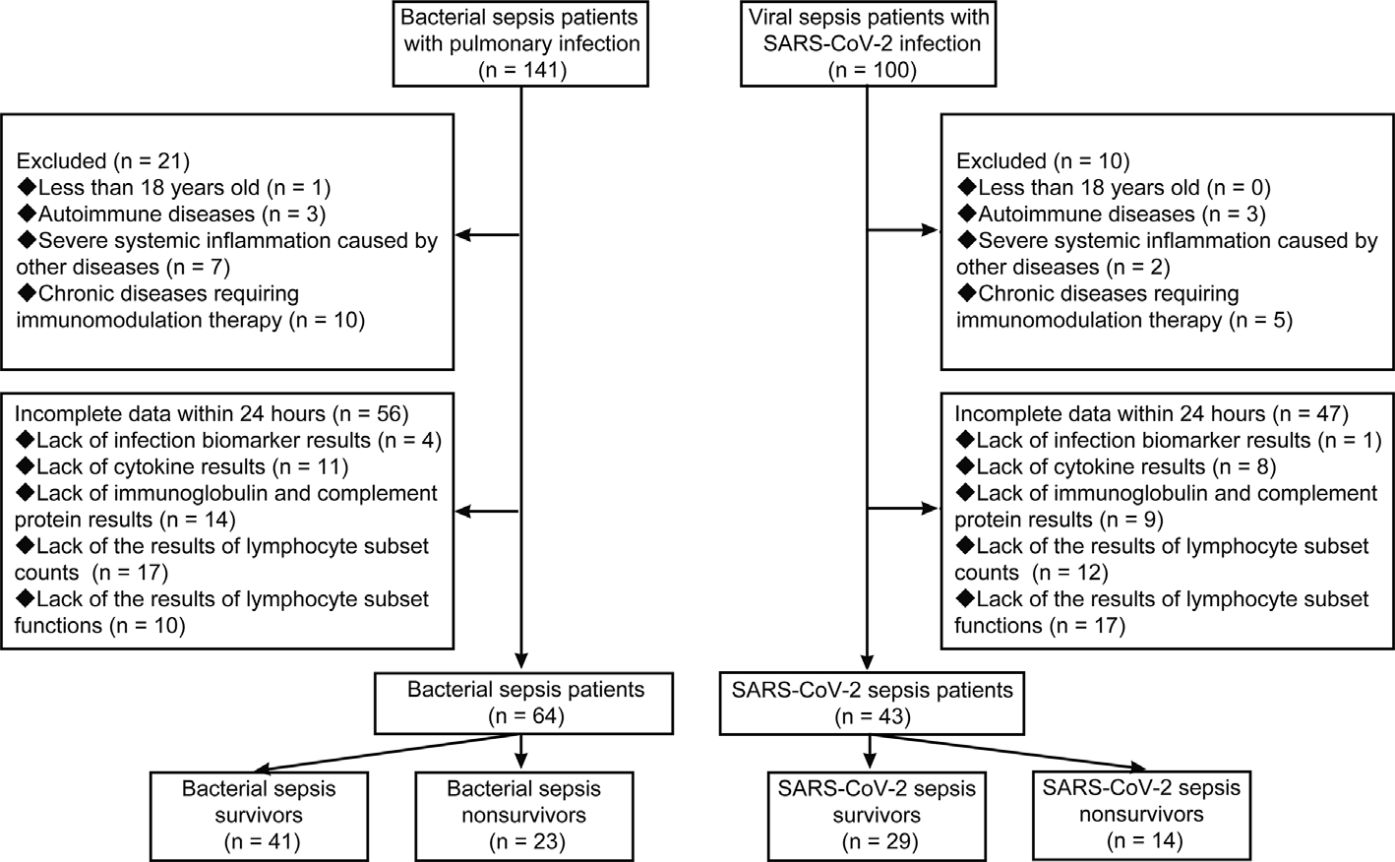
Flowchart of included and excluded patients.

A total of 64 bacterial sepsis patients with pulmonary infection and 43 SARS-CoV-2 sepsis patients were included in the present study (**Figure 1**). Gram-negative bacteria were the most common organism of infection in the bacterial sepsis group. The baseline characteristics of the enrolled patients are shown in **Table 1**. The two groups were matched with regard to age, sex, SOFA score, APACHE II score, and chronic medical illness. Patients were further divided into two subgroups: survivors and nonsurvivors according to the prognosis. Nonsurvivors were older and had higher SOFA and APACHE II scores than survivors in both the bacterial sepsis and SARS-CoV-2 sepsis groups. Differences in immune system changes between bacterial sepsis patients and SARS-CoV-2 sepsis patients, bacterial sepsis survivors and SARS-CoV-2 sepsis survivors, bacterial sepsis nonsurvivors and SARS-CoV-2 sepsis nonsurvivors, bacterial sepsis survivors and nonsurvivors, as well as SARS-CoV-2 sepsis survivors and nonsurvivors, were then compared.

**Table 1:** Baseline characteristics of patients with bacterial sepsis and SARS-CoV-2 sepsis.

	Bacterial sepsis			SARS-CoV-2 sepsis		
	Total	Survivors	Nonsurvivors	Total	Survivors	Nonsurvivors
	(n = 64)	(n = 41)	(n = 23)	(n = 43)	(n = 29)	(n = 14)
Age^a,b^, years	58.0 (51.0, 63.0)	54.0 (50.0, 62.0)	61.0 (57.0, 66.0)	57.0 (50.0, 68.0)	53 (48.5, 63.0)	63.5 (59.0, 71.0)
Age range^a,b^, years						
20–39	3 (4.7)	3 (7.3)	0	2 (4.7)	2 (6.9)	0
40–59	32 (50.0)	24 (58.5)	8 (34.8)	21 (48.8)	18 (62.1)	3 (21.4)
≥ 60	29 (45.3)	14 (34.1)	15 (65.2)	20 (46.5)	9 (31.0)	11 (78.6)
Female	23 (35.9)	15 (36.6)	8 (34.8)	14 (32.6)	10 (34.5)	4 (28.6)
SOFA score^a,b^	5.5 (4.5, 7.0)	4.0 (3.0, 6.0)	6.5 (5.0, 8.0)	5.0 (4.0, 7.0)	4.5 (3.0, 5.0)	6.0 (4.5, 8.0)
APACHE II score^a,b^	16.0 (12.0, 20.0)	14.5 (11.0, 18.5)	20.0 (16.0, 22.5)	17.0 (14.0, 18.5)	16.0 (13.5, 17.0)	19.0 (16.0, 20.0)
Chronic medical illness						
Hypertension	13 (20.3)	7 (17.1)	6 (26.1)	10 (23.3)	6 (20.7)	4 (28.6)
Chronic obstructive pulmonary disease	9 (12.5)	5 (12.2)	4 (17.4)	3 (7.0)	2 (6.9)	1 (7.1)
Diabetes mellitus	7 (10.9)	5 (12.2)	2 (8.7)	5 (11.6)	3 (10.3)	2 (14.3)
Coronary artery disease	2 (3.1)	1 (2.4)	1 (4.3)	1 (2.3)	0	1 (7.1)
Cerebrovascular disease	1 (1.6)	1 (2.4)	0	0	0	0

Data are shown as median (interquartile range) and number (percentage). ^a^Bacterial sepsis survivors vs. nonsurvivors is statistically significant. ^b^SARS-CoV-2 sepsis survivors vs. nonsurvivors is statistically significant. SARS-CoV-2, severe acute respiratory coronavirus 2; SOFA, Sequential Organ Failure Assessment; APACHE II, Acute Physiology and Chronic Health Evaluation II. Statistics obtained from chi-square tests and Fisher exact probability test.

### Blood Routine and Infection Biomarker Results Were Similar for Both Types of Sepsis

**Figure 2:**
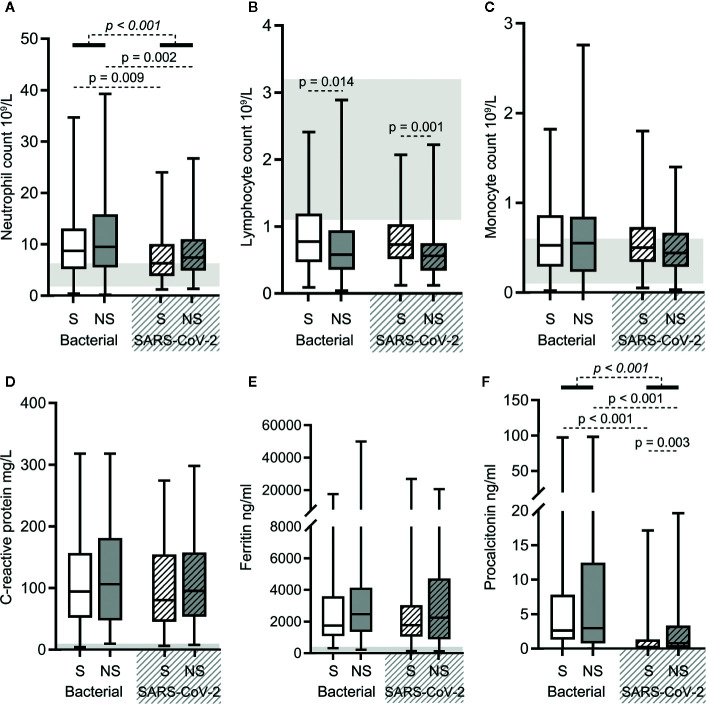
The results of blood routine and infection biomarkers in bacterial sepsis and severe acute respiratory syndrome coronavirus 2 (SARS-CoV-2) sepsis patients. Comparison of the neutrophil **(A)**, lymphocyte **(B)**, and monocyte **(C)** counts, and the levels of C-reactive protein **(D)**, ferritin **(E)**, and procalcitonin **(F)** in the two types of sepsis patients. The *p*-value of the comparison between bacterial sepsis and SARS-CoV-2 sepsis groups is shown as *italics*, and the p-value of the comparison among the survivor (S) and nonsurvivor (NS) subgroups is shown as regular. Shaded region showing the normal range of the indicated index. Normal range of procalcitonin levels (<0.05 ng/ml) is too small to be clearly displayed in the figure **(F)**. Bacterial represents the bacterial sepsis group (n = 64, S: n = 41, NS: n = 23). SARS-CoV-2 represents the SARS-CoV-2 sepsis group (n = 43, S: n = 29, NS: n = 14). Statistics obtained from the Wilcoxon rank-sum test.

Overall, the results of blood routine (neutrophil, lymphocyte, and monocyte counts) and infection biomarkers (C-reactive protein, ferritin, and procalcitonin levels) in SARS-CoV-2 sepsis patients and bacterial sepsis patients showed the same upward and downward trend relative to normal ranges (**Figure 1**). Neutrophil counts and procalcitonin (PCT) levels increased more significantly in bacterial sepsis patients, which is consistent with the characteristics of bacterial infections (**Figures 2A, F**). PCT levels were higher in SARS-CoV-2 sepsis nonsurvivors than in survivors, which was due to secondary bacterial infections in some nonsurvivors (**Figure 2F**). Lymphocyte and monocyte counts, C-reactive protein and ferritin levels did not differ significantly between the two sepsis groups (**Figures 2B–E**). In addition, there was a difference in lymphocyte counts between bacterial sepsis survivors and nonsurvivors, as well as between SARS-CoV-2 sepsis survivors and nonsurvivors (**Figure 2B**). Taken together, the two types of sepsis were similar in terms of blood routine and infection biomarker results, except for neutrophil counts and PCT levels.

### Cytokine Storm Was Milder in SARS-CoV-2 Sepsis

**Figure 3:**
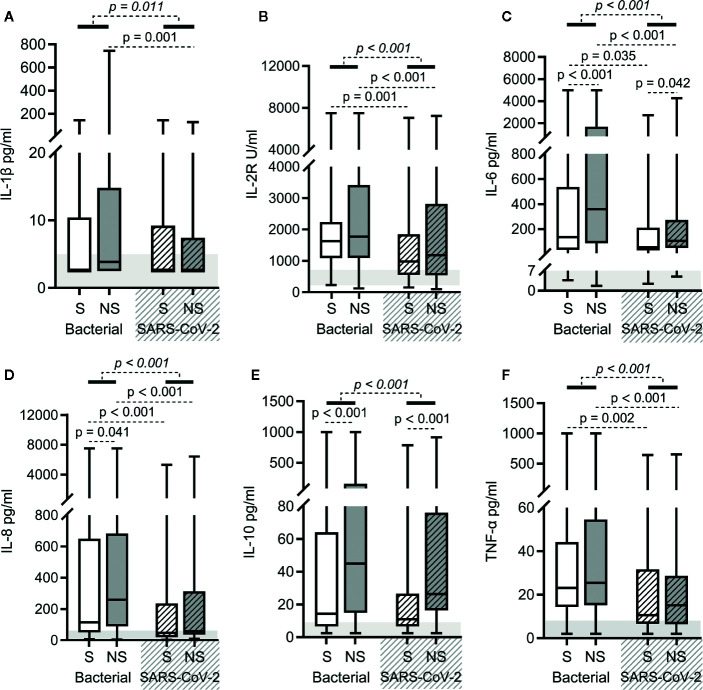
Cytokine levels in bacterial sepsis and severe acute respiratory syndrome coronavirus 2 (SARS-CoV-2) sepsis patients. Comparison of the levels of IL-1β **(A)**, IL-2R **(B)**, IL-6 **(C)**, IL-8 **(D)**, IL-10 **(E)**, and TNF-α **(F)** between bacterial sepsis and SARS-CoV-2 sepsis patients. The *p*-value of the comparison between bacterial sepsis and SARS-CoV-2 sepsis groups is shown in *italics*, and the p-value of the comparison among the survivor (S) and nonsurvivor (NS) subgroups is shown as regular font. The shaded region indicates the normal range of the indicated index. Bacterial represents the bacterial sepsis group (n = 64, S: n = 41, NS: n = 23). SARS-CoV-2 represents the SARS-CoV-2 sepsis group (n = 43, S: n = 29, NS: n = 14). Statistics obtained from the Wilcoxon rank-sum test.

As shown in **Figure 2**, elevated levels of serum cytokines (interleukin [IL]-1β, IL-2R, IL-6, IL-8, IL-10, and tumor necrosis factor [TNF]-α) were observed in both bacterial sepsis and SARS-CoV-2 sepsis groups, but were lower in the latter group than in the former (the *p*-values in italics are all < 0.05 in **Figures 3A–F**). When survivors of the two groups were compared, the levels of serum IL-2R, IL-6, IL-8, and TNF-α were lower in SARS-CoV-2 sepsis survivors. When nonsurvivors of the two groups were compared, the levels of serum IL-1β, IL-2R, IL-6, IL-8, and TNF-α were also lower in SARS-CoV-2 sepsis nonsurvivors (**Figures 3A–F**). Collectively, the cytokine storm was more moderate in SARS-CoV-2 sepsis. Besides, the levels of IL-6, IL-8, and IL-10 in bacterial sepsis nonsurvivors and the levels of IL-6 and IL-10 in SARS-CoV-2 sepsis nonsurvivors were higher when compared with the corresponding survivors (**Figures 3C–E**).

### Immunoglobulin and Complement Protein Levels Were Higher in SARS-CoV-2 Sepsis

**Figure 4:**
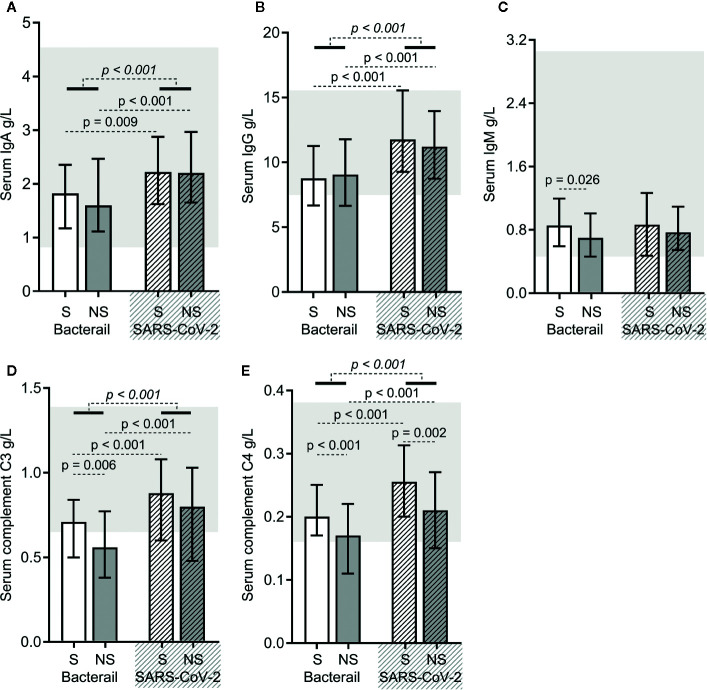
The immunoglobulin and complement protein levels in bacterial sepsis and severe acute respiratory syndrome coronavirus 2 (SARS-CoV-2) sepsis patients. The levels of immunoglobulin A (IgA) **(A)**, immunoglobulin G (IgG) **(B)**, immunoglobulin M (IgM) **(C)**, complement C3 **(D)**, and complement C4 **(E)** were compared between the two types of sepsis patients. The *p*-value of the comparison between bacterial sepsis and SARS-CoV-2 sepsis groups is shown in *italics*, and the p-value of the comparison among the survivor (S) and nonsurvivor (NS) subgroups is shown as regular font. Shaded regions showing the normal range of the indicated index. Bacterial represents the bacterial sepsis group (n = 64, S: n = 41, NS: n = 23). SARS-CoV-2 represents the SARS-CoV-2 sepsis group (n = 43, S: n = 29, NS: n = 14). Statistics obtained from the Wilcoxon rank-sum test.

As shown in **Figure 3**, except for immunoglobulin M (IgM) levels, the levels of immunoglobulin A (IgA), immunoglobulin G (IgG), complement C3, and C4 were higher in SARS-CoV-2 sepsis patients. The same was true for both the SARS-CoV-2 sepsis survivor and nonsurvivor subgroups (**Figures 4A–E**). Moreover, we found differences in the levels of IgM, complement C3 and C4 between bacterial sepsis survivors and nonsurvivors (**Figures 4C–E**), while there only differences in complement C4 levels between SARS-CoV-2 sepsis survivors and nonsurvivors (**Figure 4E**).

### SARS-CoV-2 Sepsis and Bacterial Sepsis Shared Alterations in Lymphocyte Subset Counts and Functions Except for Slight Differences

**Figure 5:**
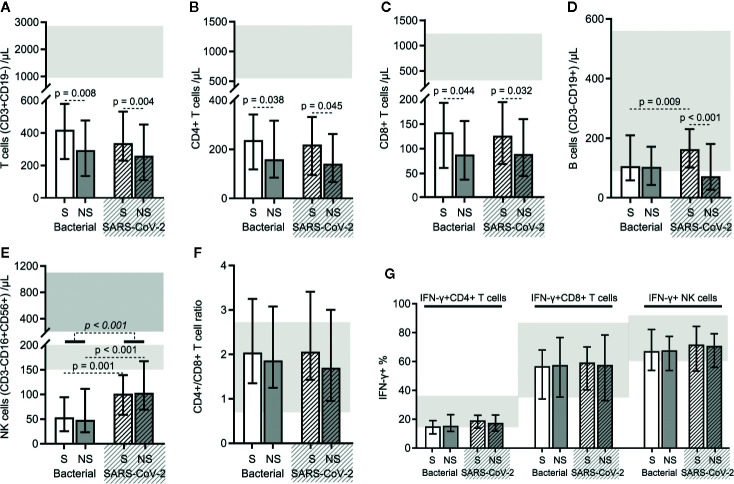
The results of the lymphocyte subset counts and functions in bacterial sepsis and severe acute respiratory syndrome coronavirus 2 (SARS-CoV-2) sepsis patients. The counts of total T lymphocytes **(A)**, CD4+ T cells **(B)**, CD8+ T cells **(C)**, B cells **(D)**, and NK cells **(E)**, and the CD4+/CD8+ T cell ratios **(F)** were compared between the two types of sepsis patients. The proportions of PMA/ionomycin-stimulated IFN-γ positive cells in CD4+, CD8+ T cells, and NK cells were also compared between the two types of sepsis patients **(G)**. The *p*-value of the comparison of bacterial sepsis and SARS-CoV-2 sepsis groups is shown in *italics*, and the p-value of the comparison of the survivor (S) and nonsurvivor (NS) subgroups is shown as regular. Shaded region showing the normal range of the indicated index. Bacterial represents the bacterial sepsis group (n = 64, S: n = 41, NS: n = 23). SARS-CoV-2 represents the SARS-CoV-2 sepsis group (n = 43, S: n = 29, NS: n = 14). Statistics obtained from the Wilcoxon rank-sum test.

Reduction in the counts of all lymphocyte subsets (total T cells, CD4+ T cells, CD8+ T cells, B cells, and NK cells) were common for both bacterial sepsis and SARS-CoV-2 sepsis groups (**Figures 5A–E**). There were no significant differences in the total T cell, CD4+ T cell, CD8+ T cell, or B cell counts, and the CD4+/CD8+ T cell ratios between the two types of sepsis groups (**Figures 5A–D, F**). NK cell counts were higher in the SARS-CoV-2 sepsis patients than in bacterial sepsis patients, as for their survivor and nonsurvivor subgroups (**Figure 5E**). Besides, the total T cell, CD4+ T cell, and CD8+ T cell counts, and the CD4+/CD8+ T cell ratios did not differ between survivors of the two types of sepsis groups (**Figures 5A–C, F**). The total T cell, CD4+ T cell, CD8+ T cell, and B cell counts, and the CD4+/CD8+ T cell ratios did not differ between nonsurvivors of the two types of sepsis groups (**Figures 5A–D, F**). Furthermore, differences were found in the total T cell, CD4+ T cell, and CD8+ T cell counts between bacterial sepsis survivors and nonsurvivors (**Figures 5A–C**). Similar results were also observed between survivors and nonsurvivors of the SARS-CoV-2 sepsis group, with the only discrepancy being a difference in B cell counts (**Figures 5A–D**). These results indicated that the difference in lymphocyte subset counts was not obvious between the two types of sepsis. Additionally, the proportions of PMA/ionomycin-stimulated IFN-γ positive cells in CD4+, CD8+ T cells, and NK cells showed no significant difference between the two types of sepsis groups, nor for the survivor and nonsurvivor subgroups (**Figure 5G**). That is, the changes in lymphocyte subset functions were also similar in the two types of sepsis.

## Discussion

A large number of patients have died of COVID-19, and the death toll is still growing rapidly. It is not difficult to imagine that almost all of these dead patients experienced organ dysfunction caused by SARS-CoV-2 infection. This is in line with the latest definition of sepsis (2). A better understanding of how the immune system is affected is an inescapable topic for the management of sepsis. In this study, we compared the immune system alterations of bacterial sepsis and SARS-CoV-2 sepsis patients. Both similarities and differences were presented. The similarities lay in the results of blood routine, infection biomarkers, and lymphocyte subset counts and functions. Differences were that in SARS-CoV-2 sepsis, the cytokine levels were lower, and the immunoglobulin and complement protein levels were higher. These similarities and differences provide important information about appropriate immunotherapy.

The cytokine storm has been widely reported to occur in severe COVID-19 and bacterial sepsis and many studies have shown that elevated levels of some cytokines, such as IL-6 and IL-10, are associated with mortality (13–17). Consistent with previous studies, elevated cytokine levels were common in the two types of sepsis of our study and there were also differences in the levels of IL-6 and IL-10 between survivors and nonsurvivors of the two sepsis groups. Notably, we found that the cytokine storm in SARS-CoV-2 sepsis was milder than that in bacterial sepsis, which is similar to the findings of Kox et al (18). For many years, bacterial sepsis mortality was considered to be secondary to an exaggerated systemic inflammatory response. Given the widespread rise in cytokine levels, it has been proposed that mortality of SARS-CoV-2 sepsis patients may be reduced by suppressing inflammatory responses to prevent the cytokine storm syndrome.

A recent study showed that dexamethasone reduced the 28-day mortality in COVID-19 patients who received either invasive mechanical ventilation or oxygen-only support (19). Although these results are encouraging, there are a few issues that merit our attention. First, dexamethasone exerts many effects, and this clinical trial has not elucidated the exact mechanism responsible for the reduction in mortality, thus it is not clear whether its activity is that of suppressing the cytokine storm. It cannot be ruled out that the main effect of dexamethasone may be to inhibit the local inflammatory reaction in the lung, which causes severe lung injury observed in these patients, and the plasma levels of inflammatory factors cannot represent the degree of lung injury. Second, dexamethasone is a broad-spectrum immunosuppressant and can interfere with the normal function of T cells, B cells, and macrophages (20). Some patients who received ventilator support in this study may have already been immunocompromised, and in theory, it may not be appropriate to further suppress the immune response. An equilibrium that can benefit patients while avoiding excessive immunosuppression needs to be found. Third, cytokines are part of a well-maintained innate immune response and are necessary for the effective elimination of infectious pathogens. Most mediators related to cytokine storms exhibit pleiotropic downstream effects often with interdependent biological activities (21). Elevated cytokines may be a biomarker of the severity of the disease, rather than a pathogenic mediator (22); thus, blind intervention may worsen an already compromised immune system. Lastly, glucocorticoids have been studied previously in animal models and clinical trials of different coronavirus infections, but the results have not been satisfactory. Glucocorticoid therapy was also shown to be beneficial to patients in the earliest study of the severe acute respiratory syndrome coronavirus (SARS-CoV) outbreak in 2003, but a recent meta-analysis pointed out that glucocorticoid therapy for SARS-CoV, the Middle East respiratory syndrome coronavirus (MERS-CoV), and SARS-CoV-2 may lead to delayed virus clearance and failure to improve the prognosis of patients (23, 24). Similarly, in studies investigating bacterial sepsis over the past few decades, sepsis researchers initially had great expectations in the therapeutic effects of suppressing the cytokine storm, but they eventually found that this approach did not improve prognosis and, in some cases, even worsened outcomes (25–28). Considering these past results and the milder cytokine storm observed in SARS-CoV-2 sepsis, we suggest that treatments involving the suppression of the immune system with the aim of counteracting the increased cytokine concentrations be administered cautiously.

IgM levels, which are closely related to the prognosis of bacterial sepsis, and their protective role in bacterial sepsis has been demonstrated repeatedly in previous studies (29–32); however, no difference in IgM levels was found in SARS-CoV-2 sepsis survivors and nonsurvivors in our study. This suggests that the decrease in IgM levels may not necessarily be associated with the prognosis of SARS-CoV-2 sepsis. Further study is necessary to investigate the underlying mechanism behind this difference. Moreover, we found IgA and IgG levels were higher in SARS-CoV-2 sepsis patients than those in bacterial sepsis patients. It should also be noted that immunoglobulins are not recommended in the treatment of bacterial sepsis (33). Thus, the use of immunoglobulins to treat SARS-CoV-2 sepsis may be also unnecessary. Our data support the weak recommendation in the Guidelines on the Management of Critically Ill Adults with COVID-19 not to routinely use standard intravenous immunoglobulins (6). Furthermore, bacterial sepsis is known to cause robust complement activation, which plays a deleterious role (34). There is also some convincing evidence available from reports describing complement activation in COVID-19 patients (35, 36). Endothelial dysfunction, coagulopathy, and cardiovascular dysregulation due to complement activation have been reported in both bacterial sepsis and COVID-19 patients (34, 37–39). Inhibition of the complement system has been shown to be beneficial in bacterial sepsis (40–42). In our study, we found there were higher levels of C3 and C4 in SARS-CoV-2 sepsis patients, thus we believe that the treatment of inhibiting hyperactivation of the complement system in SARS-CoV-2 sepsis may be worthy of further study.

It is now appreciated that sepsis leads to severe immunosuppression, which is closely related to prognosis (43–45). Massive lymphocyte depletion and dysfunction are important causes of immunosuppression (46–48). In our study, we observed that the counts of total lymphocytes, total T cells, CD4+ T cells, CD8+ T cells, and B cells, as well as the proportions of PMA/ionomycin-stimulated INF-γ positive CD4+, CD8+ T cells, and NK cells, did not differ significantly between the two types of sepsis. These results suggest that SARS-CoV-2 sepsis may share an immunosuppressive mechanism resulting from lymphocyte compartment abnormalities with bacterial sepsis. Furthermore, we also found that the counts of total lymphocytes and those of lymphocyte subsets presented a marked decline relative to their normal ranges in both types of sepsis. Lymphocytes play a central role in the anti-infective immune response due to their ability to interact with cells of the innate immune system and other cells of adaptive immunity. They are not only passive bystanders but also play a key role in the proper regulation of the inflammatory response (49, 50). In previous studies, it has been confirmed that lymphocyte depletion will worsen the prognosis of bacterial sepsis patients, and methods to alleviate lymphocyte depletion have achieved positive results in many bacterial sepsis animal studies (50–56). Therefore, we believe exploring treatments able to reduce lymphocyte consumption is warranted. In fact, immunosuppression caused by lymphopenia is also a characteristic present in many other critically ill patients, such as those experiencing severe trauma and burns (49, 57). In contrast to the rapid and transitory cytokine storm, immunosuppression is often long-term, progressive, and ultimately fatal (10). Finding a solution to lymphocyte depletion may relieve the immunosuppressive state and benefit a variety of critical illnesses, including SARS-CoV-2 sepsis.

Limitations of this study should be noted. First, although Gram-negative bacteria were the most common organism of infection in the bacterial sepsis group, we have not unified the pathogens of bacterial infections, and there may be some differences in the sepsis patterns induced by different bacterial pathogens. Second, we could confirm whether the cause of sepsis is bacterial or viral infection, although some SARS-CoV-2 sepsis patients were also complicated by bacterial infection, which may exacerbate the impact on their immune system. However, even in this context, the changes observed in the serum levels of cytokines and immunoglobulins are still milder than those observed in bacterial sepsis. Third, although all data were obtained within 24 h after the patient was diagnosed with sepsis, the uneven course of disease prior to the onset of sepsis may affect the outcome. Moreover, the immune system fluctuates widely, and the inclusion of data at multiple time points would make the conclusions more reliable. However, due to the cost of certain laboratory tests, most of our patients received those expensive examinations within 24 h of being diagnosed with sepsis. Despite these limitations, in light of previous research results and management guidelines on bacterial sepsis, our findings provide theoretical insights into the immunotherapy of SARS-CoV-2 sepsis. Within 24 h of SARS-CoV-2 sepsis, compared with bacterial sepsis, the cytokine storm is milder, immunoglobulin and complement protein levels are higher, and the lymphocyte subset counts and functions are similar. These data do not support the treatment of blocking the cytokine storm and supplementing immunoglobulins in SARS-CoV-2 sepsis, at least in the early stages of the disease. Besides, solutions aimed at hyperactivation of the complement system and lymphocyte depletion may be worthy of further investigation.

## Data Availability Statement

The raw data supporting the conclusions of this article will be made available by the authors, without undue reservation.

## Ethics Statement

The studies involving human participants were reviewed and approved by Medical Ethics Committee of Tongji Hospital, Tongji Medical College, Huazhong University of Science and Technology, Wuhan, China. Written informed consent for participation was not required for this study in accordance with the national legislation and the institutional requirements.

## Author Contributions

XD, XB and ZL conceptualized and designed the study. XD, CW, and XL acquired the data. XD and WG analyzed and interpreted the data. XD, CW, and XL drafted the article. XD, WG, XB, and ZL critically revised the manuscript. All authors contributed to the article and approved the submitted version.

## Funding

This work was supported by the National Natural Science Foundation of China (Nos. 81571891 and 81772129).

## Conflict of Interest

The authors declare that the research was conducted in the absence of any commercial or financial relationships that could be construed as a potential conflict of interest.

## Abbreviations

APACHE II score, Acute Physiology and Chronic Health Evaluation II score; COVID-19, coronavirus disease 2019; IgA, immunoglobulin A; IgG, immunoglobulin G; IgM, immunoglobulin M; NS, nonsurvivor; PCT, procalcitonin; qSOFA, quick Sequential Organ Failure Assessment; S, survivor; SARS-CoV-2, severe acute respiratory syndrome coronavirus 2.
